# Convulsions in Infancy and Childhood

**Published:** 1905-02-18

**Authors:** 


### CONVULSIONS IN INFANCY AND CHILDHOOD.

In a lecture on this subject Dr. Henry Ashby[1] says that instability of reflex centres with consequent excessive susceptibility to stimulation exists in greater or less degree in all infants and young children, but the degree of this susceptibility varies widely in different individuals. In some families there is a neurotic inheritance, and in such therefore convulsions are easily provoked. Rickets is also a condition which predisposes to convulsions, perhaps as the result of indigestion and of the absorption of toxins from the intestine. The exciting causes may be classed as (1) *reflex*, the stimulus taking origin in the gastro-intestinal tract as in griping and colic, in the respiratory passages as in pertussis, in the ear, nose or mouth—painful dentition, for example; (2) *toxic*, as in the early stage of measles or influenza; (3) *central*, as in meningitis or in syphilis of the brain. In some cases no cause can be found, and the convulsions, which are apt to be associated with evidences of defective intelligence, can only be termed *epileptiform*. In the newly-born convulsions are most frequently caused by gastro-intestinal disturbances due to artificial feeding, though in some instances they may be manifestations of epilepsy or be the result of injuries to the brain during parturition. Even a unilateral distribution of the convulsions is not inconsistent with a reflex causation, and the same may be said of a subsequent slight paresis, though a definite hemiplegia necessarily suggests organic disease. In the majority of cases in young infants, the distention of the abdomen with gas, the evidences of griping, the unhealthy character of the stools, and the excoriation of the nates indicate the point of initial irritation. Such conditions are usually found in artificially-fed children and mean infection from a contaminated food supply. The short-cut cure is a wet nurse, or failing this, asses' milk, or whey made from clean fresh cows' milk, and diluted with a solution of milk sugar; and whatever artificial method of feeding is adopted scrupulous care and cleanliness are essential. Convulsions during the period of the primary dentition are frequently, but probably with great exaggeration, referred to the eruption of the teeth, but here again feeding, or rather over-feeding with cows' milk, is the common cause, though in individual instances, nasal, pharyngeal, or bronchial catarrh, or middle-ear suppuration may be responsible. In the form of convulsions known as child-crowing, or laryngismus stridulus, the adductor muscles of the vocal cords are the seat of spasm. Practically this condition is always associated with rickets as a predisposing cause, whilst emotion, bronchial catarrh, or gastro-intestinal irritation may determine the occurrence of the spasm. Among causes of brain irritation leading to convulsions may be mentioned meningitis, encephalitis, thrombosis, hæmorrhage, tumours, and syphilis. In meningitis the fits may be late consequences of the disease process, but, on the other hand, they may be among the earliest symptoms. In these latter circumstances it is a difficult matter to distinguish them from reflex convulsions due, perhaps, to gastro-intestinal irritation. In each the convulsions may be more or less one-sided and may be attended with coma and retraction of the head. Syphilitic softening of the brain cortex is also apt to produce a series of slight one-sided convulsions, each accompanied by momentary unconsciousness or a temporarily dazed expression. In other children slight fits frequently recurring without apparent cause and with defective mental development suggest some failure of development of the higher centres and carry an anxious prognosis.

One question arising in connection with convulsions in childhood is the relation of these to epilepsy. In the majority of cases, especially those associated with rickets, the convulsions tend to become less and less frequent and ultimately disappear. But in some ten per cent. of those who suffer from epilepsy in later life, a history of convulsions in early life can be obtained. Hence, when no cause can be discovered for such convulsions, and more especially if at the same time there is any mental peculiarity, the outlook is not free from anxiety. The discovery of a definite cause, such as worms, dyspepsia, etc., renders tenable a reflex interpretation and so materially relieves the prognosis.

Serious convulsive attacks, usually attended with a high temperature, and recurring more or less rapidly, may be followed by signs of permanent mischief, either mental or bodily, and in these there is probably an organic brain lesion. Hemiplegia and mental weakness are the commonest consequences. Convulsions of this order may be associated with measles, pertussis, or other infective disease. In exceptional instances such evidences of brain injury, as blindness, deafness, and aphasia may ensue.

The rational treatment of any convulsive seizure is, of course, the treatment of the condition of which it is an expression; but certain measures are necessary to meet the emergency and independently of the source of irritation. During the attack the subcutaneous injection of morphine is the most powerful remedy to check the convulsive movements and this applies whether colic, meningitis, or laryngismus complicates the situation. A strong child of six months may have a fortieth of a grain, and one of twelve months a twentieth of a grain of morphine. But in newly-born or very young infants, or in those who are feeble and wasted, morphine should be avoided. Chloroform is a prompt but temporary expedient for checking convulsions. A more durable one is chloral hydrate; four or five grains dissolved in water or in egg and milk mixture may be given per rectum to children of six to twelve months. In all cases a dose of calomel is well-advised with a view to clear irritating material from the bowel. To prevent further attacks measures calculated to brace up and harden the nerve centres are needed. These include open-air discipline and not the hot, stuffy rooms in which children with laryngismus stridulus are often immured. If properly clothed the child may enjoy an outside life even in the coldest weather. The diet, too, must be carefully regulated; many infants get too much milk, and are found to have foul and pale stools.

In older children with fits admittedly epileptic or of the "borderland" class, diet, also, is of great importance. Porridge, rice puddings, bread, and other starchy substances may readily be overdone and may cause intestinal fermentation; much the same, too, may be said of milk. Cocoa, with egg and toast, for breakfast; some meat or poultry, with cooked vegetables and stewed fruits, for dinner; and cocoa and toast, with treacle or honey for tea, is the scheme adapted to most cases. If school is contra-indicated there is every need for the cultivation of self-control by a regular system of order and supervision. To allow these children to be idle and unoccupied is a sure way to exaggerate the already unstable condition of their brain centres.

1 Lancet, Jan. 21, 1905.

